# Can Characteristics of Reciprocal Translocations Predict the Chance of Transferable Embryos in PGD Cycles?

**DOI:** 10.3390/jcm3020348

**Published:** 2014-04-02

**Authors:** Elsbeth Dul, Jannie van Echten-Arends, Henk Groen, Peter Kastrop, Lucie Amory-van Wissen, John Engelen, Jolande Land, Edith Coonen, Conny van Ravenswaaij-Arts

**Affiliations:** 1 Department of Obstetrics and Gynaecology, University of Groningen, University Medical Center Groningen, Hanzeplein 1, GZ Groningen 9713, The Netherlands; E-Mails: j.van.echten@umcg.nl (J.E.-A.); j.a.land@umcg.nl (J.L.); 2 Department of Epidemiology, University of Groningen, University Medical Center Groningen, Hanzeplein 1, GZ Groningen 9713, The Netherlands; E-Mail: h.groen01@umcg.nl; 3 Department of Reproductive Medicine, University Medical Center Utrecht, Heidelberglaan 100, CX Utrecht 3584, The Netherlands; E-Mail: peter_kastrop@hotmail.com; 4 Department of Obstetrics & Gynaecology, Academic Hospital Maastricht, P. Debyelaan 25, HX Maastricht 6229, The Netherlands; E-Mails: lucie.amory@mumc.nl (L.A.W.); edith.coonen@mumc.nl (E.C.); 5 Department of Clinical Genetics, Academic Hospital Maastricht, P. Debyelaan 25, HX Maastricht 6229, The Netherlands; E-Mail: john.engelen@mumc.nl; 6 Department of Genetics, University of Groningen, University Medical Center Groningen, Hanzeplein 1, GZ Groningen 9713, The Netherlands; E-Mail: c.m.a.van.ravenswaaij@umcg.nl

**Keywords:** reciprocal translocation, Robertsonian translocation, preimplantation genetic diagnosis (PGD), meiotic segregation

## Abstract

Translocation carriers have an increased risk of miscarriage or the birth of a child with congenital anomalies. Preimplantation genetic diagnosis (PGD) is performed in translocation carriers to select for balanced embryos and, thus, increase the chance of an ongoing pregnancy. However, a common experience is that reciprocal translocation carriers produce a high percentage of unbalanced embryos, which cannot be transferred. Therefore, the pregnancy rates in PGD in this patient group are low. In a cohort of 85 reciprocal translocation carriers undergoing PGD we have searched for cytogenetic characteristics of the translocations that can predict the percentage of balanced embryos. Using shape algorithms, the most likely segregation mode per translocation was determined. Shape algorithm, breakpoint location, and relative chromosome segment sizes proved not to be independent predictors of the percentage of balanced embryos. The ratio of the relative sizes of the translocated segments of both translocation chromosomes can give some insight into the chance of transferable embryos: Very asymmetrical translocations have a higher risk of unbalanced products (*p* = 0.048). Counseling of the couples on the pros and cons of all their reproductive options remains very important.

## 1. Introduction

Since 1997 preimplantation genetic diagnosis (PGD) has been performed worldwide to allow translocation carriers to conceive balanced offspring and decrease the risk of miscarriages. However, it has become clear that reciprocal translocation carriers produce many unbalanced embryos [1], and PGD offers acceptable pregnancy rates only if the woman responds to ovarian hyperstimulation by producing many oocytes. Thus far, no prediction model for the success of a PGD treatment in translocation carriers has been postulated.

Translocations are present in 0.2% of the general (neonatal) population [2,3]. Carriers of Robertsonian and reciprocal translocations have an increased risk of a miscarriage or the birth of a child with congenital anomalies caused by an unbalanced karyotype. Furthermore, translocation carriers can suffer from infertility, which is especially true in male carriers of Robertsonian translocations [4].

To significantly reduce the chance of conceiving unbalanced offspring PGD can be performed. In this procedure, an *in vitro* fertilization (IVF) treatment is executed and the embryos are tested for their genetic make-up. Only normal or balanced embryos are transferred into the uterus. Some studies have shown that PGD is effective in reducing the number of miscarriages and children born with an unbalanced translocation in comparison to spontaneous conception [1,5,6,7]. However, other studies indicate that the live birth rate after PGD is not significantly different from spontaneous conception [8]. This might be explained by the fact that in translocation carriers, especially reciprocal translocation carriers, 67% to over 80% of embryos are found to be chromosomally abnormal [1,9,10,11]. In translocation carriers the pregnancy rate after oocyte collection is significantly lower (17%–24%) than after embryo transfer (26%–33%), reflecting the high proportion of abnormal embryos that are unsuitable for transfer [1,10].

The unbalanced products are the result of the segregation of the chromosomes involved in the translocation during the first meiotic cell division of gametogenesis. Reciprocal translocation chromosomes pair their homologous segments, forming a quadrivalent figure, which can segregate in five different ways: alternate (producing balanced or normal gametes), adjacent-1 and adjacent-2, 3:1, and 4:0, all of which will produce unbalanced gametes [12]. Of the 32 possible segregation products, only one results in a completely normal genotype, and one in a balanced genotype of the gamete. Depending on the chromosomes involved, their size and the location of the breakpoints, translocations are prone to segregate in one or more of the five modes mentioned. The chromosomes involved in a Robertsonian translocation pair their homologous segments forming a trivalent figure, which usually undergoes 2:1 segregation, while 3:0 segregation is extremely rare. “Alternate” 2:1 segregation results in normal or balanced gametes, while adjacent segregation produces four types of unbalanced gametes. If the chromosomes involved are of approximately the same size (symmetrical Robertsonian translocation), the translocation is more prone to segregate in an alternate way than if the chromosomes are of different size (asymmetrical Robertsonian translocation) [13].

Most reciprocal translocations are private, *i.e.*, unique for the family. Thus, in contrast to Robertsonian translocations and a few recurring reciprocal translocations, for most reciprocal translocations no empirical data on the risks for unbalanced offspring exist. For each individual couple, a clinical geneticist estimates the risk of viable unbalanced offspring based on the translocation at hand with its unique breakpoints and expected segregation modes, and the family history. These estimates are based on spontaneous conceptions, without the use of assisted reproductive technology (ART) [14,15,16]. From this experience, for example, it is evident that the risk of unbalanced offspring due to 3:1 segregation is higher if the translocation carrier is female [17,18,19]. This was recently also shown for couples who underwent PGD. In this study, 3:1 segregation was three times more likely in female translocation carriers [1]. However, from studies on segregation modes in sperm of male translocation carriers it appeared that the empirical risk estimates based on live born and stillborn babies cannot be used to predict the unbalanced outcome in gametes and early pregnancy products [20]. Since the advent of PGD, studies on segregation modes in preimplantation embryos have been published [9,20,21,22]. They report differences in segregation modes according to the gender of the reciprocal translocation carrier, although these differences did not affect the proportion of balanced embryos in PGD and the take-home baby rate [10].

We hypothesized that differentiation of reciprocal translocations based on particular characteristics may be used in predicting the chance of transferable embryos in PGD cycles, and subsequent live birth rates.

## 2. Experimental Section

### 2.1. Study Subjects

We performed a retrospective cohort study in all couples in the Netherlands undergoing PGD for translocations from October, 1997, until December, 2010. Only couples that had at least four embryos biopsied were included in the study. Couples in which both partners were carriers of a translocation were excluded, as well as PGD cycles for complex translocations (*i.e.*, more than two chromosomes involved or more than two breakpoints) or translocations involving a sex chromosome. The biopsy results of 85 couples with a reciprocal translocation were compared with the results in 35 couples with a Robertsonian translocation. Before PGD treatment started, all couples gave informed consent for use of their clinical data for research purposes.

### 2.2. Chromosomal Analysis and Pachytene Shape Statistics

Structural chromosomal abnormalities were detected by routine karyotyping of lymphocytes.

All reciprocal autosomal 2-breakpoint translocations were categorized using different systems as explained in Table 1 by two authors (Elsbeth Dul, Conny van Ravenswaaij-Arts) individually.

**Table 1:** Possible categories of reciprocal translocations.

Variable	Categories/Definition	Example
Pachytene-diagram ^a^	Translocation predisposed to the adjacent-1 or adjacent-2 type disjunction	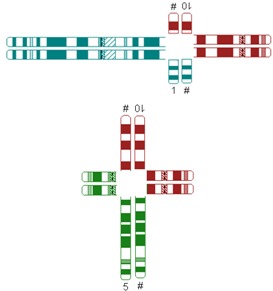
	Translocation predisposed to tertiary monosomy or trisomy (3:1 segregation)	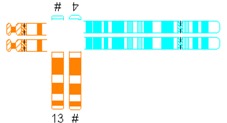
	Translocation segregating by either adjacent-1 or 3:1 type	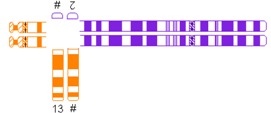
Quadrivalent ^b^	Fairly symmetric quadrivalent	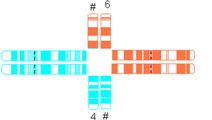
	One very small translocated segment	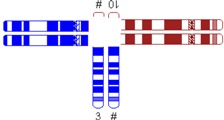
	Both translocated segments very small	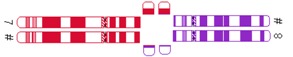
	One very small translocated segment and one very small nontranslocated segment	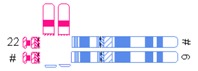
Location of breakpoints	Both in the centromeric region	
	Both in the middle of the chromosomal arm	
	Both in the telomeric region	
	Other combination	
Ratio of chromosomal length (cm)	Length of largest translocation chromosome divided by length of smallest translocation chromosome (A/B)	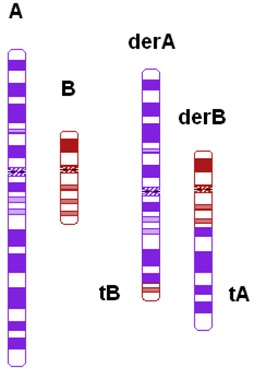
Ratio of chromosomal length with correction (cm)	Abovementioned variable with correction for heterochromatine (in chromosome 1, 9, and 16) and acrosomes	
Ratio of the relative sizes of both translocated segments ^c^	Ratio of the relative size of one translocated segment (tA/A) and the relative size of the other translocated segment (tB/B) in such a way that the largest relative size is divided by the smallest relative size (tA/A)/(tB/B)	
Total length of the translocated segments ^c^	tA + tB	

^#^ Derivative chromosome; ^a^ Based on Jalbert *et al.* [15]; ^b^ Based on Anton *et al.* [14]; ^c^ Sizes were determined in megabases (Mb) based on UCSC Genome Bioinformatics [23].

The shape algorithms used were based on Jalbert *et al.* [15], *viz.* the ratio of the sum of the centric segments to the sum of the translocated segments and the ratio of the shortest centric segment to the shortest translocated segment, and on Anton *et al.*, regarding the symmetry of the quadrivalent [14]. Quadrivalents of translocations in our database that appeared comparable to the quadrivalents shown in the publication of Anton *et al.* [14] were categorized likewise. In general, a translocation is considered to potentially have a 3:1 segregation mode if the longest axis of separation, as seen in the quadrivalent, is resulting in a 3:1 segregation [15]. This is usually the case when the quadrivalent is highly asymmetric. Breakpoint locations and relative chromosome segment sizes, including and excluding heterochromatic and variable regions, were measured using the International Standing Committee on Human Cytogenetic Nomenclature (ISCN) ideograms [24]. In order to calculate the ratio of the relative sizes of both translocated segments and the total lengths of the translocated segments, the lengths of the translocated segments and the total chromosomal lengths were determined in megabases (Mb), based on UCSC Genome Bioinformatics [23].

### 2.3. IVF and PGD

Controlled ovarian hyperstimulation was performed using a long desensitization protocol with GnRH agonists, recombinant FSH and hCG. Oocytes were retrieved transvaginally 36 h after hCG administration. Fertilization was performed by IVF or ICSI. After three days of culture, the embryos were biopsied and one or two blastomeres aspirated. Genetic analysis was performed according to the PGD guidelines [25] and probes were chosen based on the specific translocation. Embryos were scored, based on their FISH signals, as balanced, unbalanced, or inconclusive [12,26].

### 2.4. Statistical Analysis

A linear regression analysis was performed, looking for independent predictors of the percentage of balanced embryos per couple. The predictors included in the model were the gender of the translocation carrier and the classifications summarized in Table 1. Statistical significance was defined as *p* < 0.05.

## 3. Results and Discussion

PGD was performed in 85 couples with 83 different reciprocal translocations. Two times, two couples carried the same translocation. In one of these sets, both carriers were female, in the other set the gender of the carrier differed. No couples with translocations between homolog chromosomes were referred for PGD during the study period. There were 41 female carriers and 44 male carriers. As a control group we analyzed 35 couples with Robertsonian translocations: 27 symmetrical, all (13;14), and eight asymmetrical (D;G). Most of the Robertsonian translocation carriers were male (25/35).

Table 2 summarizes the results of the biopsied embryos.

**Table 2:** Results of the preimplantation genetic diagnosis (PGD) analysis of 120 couples.

Embryo Results per Couple	Reciprocal Translocations	Robertsonian Translocations
	85 Couples	35 Couples
	Median (Interquartile Range)/Couple	Median (Interquartile Range)/Couple
	{Total Number of Embryos}	{Total Number of Embryos}
Number of biopsied embryos	16 (17) {1527}	12 (8) {503}
Number of balanced embryos	2 (2) {194}	3 (4) {123}
Number of embryos with inconclusive results	1 (3) {142}	1 (2) {48}
Number of unbalanced embryos	12 (14) {1169}	7 (8) {325}
Number of excluded embryos ^a^	{22}	{7}
Percentage of balanced embryos	11.43 (12.45)	22.22 (22.84)

^a^ Embryos that were biopsied, but not included because of: haploidy, triploidy, tetraploidy; no nucleus biopsied or lysis of blastomere. This never resulted in less than four embryos biopsied per couple.

Table 3 shows the results of the univariate regression analysis of the reciprocal translocations (for the complete data of the univariate regression analysis see Supplementary Table S1).

**Table 3:** Univariate linear regression analysis for 85 reciprocal translocation carriers, regarding the percentage of balanced embryos in PGD analysis.

Variable	*N*	Median % Balanced Embryos	*p*-Value ^a^
**Type of Translocation**			
Reciprocal	85	11.43	<0.001
Robertsonian Symmetrical	27	28.00	ref
Robertsonian asymmetrical	8	15.59	0.002
**Carrier**			
Female	41	11.43	ref
Male	44	11.81	0.383
**Pachytene-Diagram**			
Adjacent-1 or 2	48	12.50	ref
3:1	14	12.50	0.534
Adjacent-1 and 3:1	23	10.00	0.846
**Quadrivalent**			
Symmetrical	23	11.11	ref
1 small translocated segment	22	13.03	0.354
Both translocated segments small	30	13.39	0.753
1 small translocated segment en 1 small nontranslocated segment	10	2.27	0.109
**Location of Breakpoints**			
Both in centromeric region	11	12.50	ref
Both in middle of arm	3	7.69	0.35
Both in telomeric region	27	13.56	0.697
Other combination	44	11.11	0.316
**Ratio of chromosomal length (cm)**	85	11.43	0.776
**Ratio of chromosomal length with correction (cm)**	85	11.43	0.505
**Ratio of the relative sizes of both translocated segments**	85	11.43	0.048
**Total length of the translocated segments (Mb)**	85	11.43	0.561

^a^ *p*-Value of category compared to the reference category; ref = reference category.

The type of translocation was an independent predictor for the percentage of balanced embryos per couple. Both reciprocal and asymmetrical Robertsonian translocations produced significantly less balanced embryos than symmetrical Robertsonian translocations. There was no significant difference in the percentage of balanced embryos between male and female reciprocal translocation carriers. None of the studied classification systems showed a significant relation with the percentage of balanced embryos per couple at PGD analysis, except for the ratio of the relative sizes of the translocated segments (*p*-value = 0.048). This shows that with a higher ratio, the chance of balanced embryos declines. This indicates that if the relative translocation segment sizes are very different from each other, the percentage of unbalanced embryos increases.

In this study, we focused on the part of the PGD trajectory from oocyte retrieval to embryo transfer, as that is the success-limiting part in PGD for reciprocal translocations. The chance of an ongoing pregnancy after embryo transfer is comparable to PGD for other genetic indications [10]. Unfortunately, we had no information on the reason why karyotyping was performed in the couples. It is likely that the most frequent indication was recurrent miscarriage, though a previous child with an unbalanced translocation, a familial translocation, or infertility might have been indications. Infertility might influence the frequency of balanced gametes, as might be concluded from the offspring and PGD results in male carriers of a Robertsonian translocation [27,28,29]. An increased frequency of infertility is seen in male carriers of Robertsonian translocation (13;14) and they have more balanced offspring, both in contrast to female carriers of this Robertsonian translocation. However, such a relation has not been described for reciprocal translocations. X-Autosome translocations can be associated with infertility, but we excluded translocations with sex chromosomes from this study.

The size of the translocated segments, calculated as the sum of the lengths from the telomeres to the breakpoints, did not influence the percentage of balanced embryos. One might hypothesize that this variable is a predictor of viability of unbalanced offspring, but that was not the subject of our study. On the other hand, a very large total length of translocated segments might result in embryos not reaching the biopsy stage of PGD, thus resulting in more balanced embryos and a positive correlation between total length and percentage of balanced embryos. Such an association was not found.

Research into segregation modes of translocation chromosomes in cleavage stage embryos is scarce, and studies consist of small numbers of embryos. Thus far, none of the studies have made clear whether the quadrivalent figure or the sizes of the translocated segments can predict the chance of balanced embryos during a PGD procedure. It is quite possible that other research into this subject has been undertaken, but not published, due to the unpredictability of translocations.

With this study, we have tried to find cytogenetic predictors for the percentage of balanced embryos in reciprocal translocation carriers. We hypothesized that more unbalanced embryos are produced in translocations that are more asymmetrical. A theoretical explanation for this might be that the alternate segregation, resulting in normal and balanced gametes, is more likely to occur in symmetric quadrivalents. Therefore, we expected the categories that reflect asymmetry in the variables “pachytene-diagram” and “quadrivalent” to produce more unbalanced embryos. However, in these variables, we did not find a significant difference between asymmetrical and symmetrical quadrivalents. This may be caused by the small numbers in the different categories of these variables. The calculation of the ratio between the exchanged segments of the translocation is more objective and reproducible. Furthermore, it is a linear parameter, which means that the cohort can be analyzed as a whole. In the present study, this calculated ratio of the relative sizes of the translocated segments is the only predictor of success in PGD in reciprocal translocation carriers. This ratio suggests that with a more asymmetrical quadrivalent, the chance of balanced embryos declines. By performing a multivariate regression analysis, the stepwise removal of non-significant factors has been evaluated. No other independent predictors of balanced embryos were found. Whether the ratio of translocated segments is indeed a good predictor of balanced embryos has to be studied in larger cohorts, preferably by using international databases, such as the European Society of Human Reproduction and Embryology (ESHRE) PGD Consortium data.

Another reason for not finding a correlation between the percentage of balanced embryos and the two ways of predicting the segregation mode of the translocation can be that the prediction models of Jalbert [15] and Anton [14] are based on live born and stillborn babies. Their categorizing of a translocation into a certain segregation mode, thus, does not exclude that other segregation modes contribute to the chromosomal make-up of the gametes of the carrier.

As the percentage of balanced embryos in our study was low (11.4%), we could only perform a univariate analysis. Therefore we could not formulate a prediction model for the chance of balanced embryos per individual translocation. The percentage of balanced embryos found in our study is in accordance with the data collection from the ESHRE PGD Consortium [10]. Larger groups of embryos are necessary to allow for multivariate analysis into the relation between the type of reciprocal translocation and the percentage of balanced embryos.

## 4. Conclusions

We have shown that the number of transferable embryos in PGD treatment in reciprocal translocation carriers is difficult to predict from the characteristics of the individual translocations. The ratio of the relative sizes of the translocated segments of both chromosomes involved in the translocation can give some insight into the chance of transferable embryos. The implications are that for translocation carriers the pregnancy rates per PGD cycle are low, irrespective of the translocation characteristics, due to a high number of untransferable embryos. Further studies are needed to establish whether the ratio of translocated segments can be used in the PGD counseling of couples. A sufficient response to oocyte stimulation remains an important requisite to increase the chance of transferable embryos. The counseling of couples with translocations remains very important, so they can decide which reproductive option (PGD, gamete donation, or natural conception) suits them best.

## Supplementary Files

Supplementary File 1
