# Supplementary material for: Can Characteristics of Reciprocal Translocations Predict the Chance of Transferable Embryos in PGD Cycles?
Source: J Clin Med. 2014 Apr 2;3(2):348–58. doi: 10.3390/jcm3020348 (PMC4449682; doi:10.3390/jcm3020348)
Supplement: Supplementary File 1 [file jcm-03-00348-s001.pdf]

# Supplementary Information

**Table S1.** Univariate linear regression analysis for 85 reciprocal translocation carriers, regarding the percentage of balanced embryos in PGD analysis.

| Variable | *N* | *R* | *R* Square | *B* | St Error | *p* | Median % Balanced Embryos |
|---|---|---|---|---|---|---|---|
| **Type of Translocation** | | 0.551 | 0.303 | | | | |
| Reciprocal | 85 | | | −17.125 | 2.402 | <0.001 | 11.43 |
| Robertsonian symmetrical | 27 | | | ref | | | 28.00 |
| Robertsonian asymmetrical | 8 | | | −13.993 | 4.376 | 0.002 | 15.59 |
| **Carrier** | | 0.96 | 0.009 | | | | |
| Female | 41 | | | ref | | | 11.43 |
| Male | 44 | | | −1.642 | 1.873 | 0.383 | 11.81 |
| **Pachytene-Diagram** | | 0.069 | 0.005 | | | | |
| Adjacent-1 or 2 | 48 | | | ref | | | 12.50 |
| 3:1 | 14 | | | −1.649 | 2.642 | 0.534 | 12.50 |
| Adjacent-1 and 3:1 | 23 | | | −0.429 | 2.206 | 0.846 | 10.00 |
| **Quadrivalent** | | 0.255 | 0.065 | | | | |
| Symmetrical | 23 | | | ref | | | 11.11 |
| 1 small translocated segment | 22 | | | 2.36 | 2.53 | 0.354 | 13.03 |
| Both translocated segments small | 30 | | | 0.744 | 2.351 | 0.753 | 13.39 |
| 1 small translocated segment en 1 small nontransloc segment | 10 | | | −5.212 | 3.214 | 0.109 | 2.27 |
| **Location of Breakpoints** | | 0.146 | 0.021 | | | | |
| Both in centromeric region | 11 | | | ref | | | 12.50 |
| Both in middle of arm | 3 | | | −5.313 | 5.654 | 0.35 | 7.69 |
| Both in telomeric region | 27 | | | −1.211 | 3.105 | 0.697 | 13.56 |
| Other combination | 44 | | | −2.95 | 2.926 | 0.316 | 11.11 |
| **Ratio chrom length (cm)** | 85 | 0.031 | 0.001 | −0.361 | 1.265 | 0.776 | 11.43 |
| **Ratio chrom length with correction (cm)** | 85 | 0.073 | 0.005 | −0.562 | 0.84 | 0.505 | 11.43 |
| **Ratio of the relative sizes of both translocated segments** | 85 | 0.215 | 0.046 | −0.226 | 0.113 | 0.048 | 11.43 |
| **Total length of the translocated segments (Mb)** | 85 | 0.064 | 0.004 | 0.012 | 0.021 | 0.561 | 11.43 |

*R* square = goodness of fit; B = regression coefficient; ref = reference category.
